# Sex differences in the impact of social relationships on individual vocal signatures in grey mouse lemurs (*Microcebus murinus*)

**DOI:** 10.1098/rstb.2023.0193

**Published:** 2024-06-23

**Authors:** Alexandra Langehennig-Peristenidou, Marina Scheumann

**Affiliations:** ^1^ Institute of Zoology, University of Veterinary Medicine Hannover, Hannover 30559, Germany

**Keywords:** primate, vocal convergence, vocal divergence, vocal learning, individual vocal signatures, genetic relatedness

## Abstract

Vocalizations coordinate social interactions between conspecifics by conveying information concerning the individual or group identity of the sender. Social accommodation is a form of vocal learning where social affinity is signalled by converging or diverging vocalizations with those of conspecifics. To investigate whether social accommodation is linked to the social lifestyle of the sender, we investigated sex-specific differences in social accommodation in a dispersed living primate, the grey mouse lemur (*Microcebus murinus*), where females form stable sleeping groups whereas males live solitarily. We used 482 trill calls of 36 individuals from our captive breeding colony to compare acoustic dissimilarity between individuals with genetic relatedness, social contact time and body weight. Our results showed that female trills become more similar the more time females spend with each other, independent of genetic relationship, suggesting vocal convergence. In contrast, male trills were affected more by genetic than social factors. However, focusing only on socialized males, increasing time as cage partners caused greater divergence in males’ trills. Thus, grey mouse lemurs show the capacity for social accommodation, with females converging their trills to signal social closeness to sleeping group partners, whereas males do not adapt or diverge their trills to signal individual distinctiveness.

This article is part of the theme issue 'The power of sound: unravelling how acoustic communication shapes group dynamics'.

## 1. Introduction

Vocalizations coordinate social interactions in animals through the information they encode [1–3]. This information is decoded by the recipients, allowing them to respond accordingly. Thus, vocalizations enable coordinated social interactions between signallers and receivers even over long distances and without visual input [4–6]. In addition to information about the physical characteristics and emotional state of the sender [7,8], vocalizations can deliver insights into the social relationship between conspecifics, which affect social interactions (e.g. socially close group members are more likely to support each other; [9,10]). Social relationships can be signalled either by selective responses of the recipient to conspecific vocalizations (e.g. vocal networks; [11]) or by similarities or dissimilarities of vocalizations between conspecifics as a result of genetic relationship [12–14], or vocal accommodation [15,16]. According to Ruch *et al*. [17], vocal accommodation constitutes a special form of vocal production learning, where animals modify existing call types depending on changes in the environment (environmental accommodation) or social experience (social accommodation).

Social accommodation describes that individuals converge (becoming more similar) or diverge (becoming less similar) the acoustic structure of existing call types between individuals [17]. Social convergence has been observed in birds and mammals (see for review [17–21]), where individuals adjust their individual vocal signatures to those of their group and/or socially close group members (e.g. [12,22,23]). Signalling group membership is important to facilitate group cohesion by increasing the speed of the recognition of group members and keeping contact with each other even over longer distances [17,20,22]. Furthermore, it allows new group members to socially integrate into an existing group [17]. Signalling individual social affinity functions in strengthening social bonds and advertising support for single group members [9,10,17,19,23]. In contrast, social divergence is used to pronounce differences between groups or individuals of the same species, mostly in relation to territorial defence [17]. In parrots, it was found that the birds’ calls diverge from playbacks depending on the familiarity of the playback donor [24–26]. In the study of Crockford *et al*. [27], this was also shown for chimpanzees, with pant hoots from neighbouring chimpanzee groups being more dissimilar to each other than to stranger groups, independent of genetic relatedness. Thus, it was argued that individuals modify their vocalizations to be different from their neighbours [28].

Social accommodation has mainly been investigated and reported in pair-bonded and group-living birds and mammals (e.g. [15,17]), whereas to our knowledge, reports for dispersed living species are rare (e.g. [29,30]). However, non-group-living species also have individual vocal signatures (e.g. [29]) that can be used to recognize individuals [6,31]. A valid argument is that dispersed or solitarily living species, which only meet during mating, may not have the same need to adjust their calls to those of their conspecifics. Furthermore, there is a trade-off between social accommodation and honest signalling of the physical attributes of the sender, which is important for mating [7,32,33]. Thus, it could be assumed that while individual vocal signatures are present irrespective of the social structure of a species, the existence of social accommodation might be dependent on the function of the call and the social lifestyle of the sender [17,24,34]. This is further supported by the fact that social accommodation mainly affects call types used in social contexts [20,35]. Therefore, in solitary-living species, individual vocal signatures may be a byproduct of anatomical differences in the vocal apparatus representing honest signals for mate quality (genetic compatibility and biological fitness [7,36,37]), whereas in social-living species, individual vocal signatures can be shaped by the social environment. Thereby, the pattern of convergence or divergence might also be dependent on the function of the call and the social organization of the species [17,26]. This was supported by a playback study in orange-fronted conures, where sex-specific differences in the response to converged or diverged playback stimuli of conspecifics were found [26]. To investigate the impact of social lifestyle and call function on social accommodation, we studied the dispersed-living grey mouse lemur. Grey mouse lemurs are a promising primate model as they combine two social lifestyles. Since females form long-term stable sleeping groups whereas males live solitarily [38,39], sex differences in social accommodation of individual vocal signatures would be expected.

Grey mouse lemurs (*Microcebus murinus*) are small, nocturnal, seasonally breeding, strepsirrhine primates endemic to Madagascar [40]. They live in a multi-male/multi-female neighbourhood showing a large overlap of their home ranges and have access to several potential mating partners [39]. Both sexes forage solitarily during the night, but during the daytime, females form long-term stable sleeping groups, whereas males sleep mostly alone [39]. Female sleeping groups have a matrilineal grouping pattern [38] and show cooperative infant care, with communal nursing and adoption of infants of deceased female group members having been observed [41]. Whereas the females stay with their mother (female philopatry), males disperse from their natal group at around seven months (before the next mating season) [42]. Grey mouse lemurs possess a broad vocal repertoire, consisting of at least 10, mostly ultrasonic, call types (e.g. [43,44]) and have already been shown to have the capacity for environmental accommodation (Lombard effect: [45]). The most complex call type is the trill call, which serves a sex-specific social function [46,47]. Males emit trills during mating to advertise their reproductive fitness and acquire access to mating partners [46,48,49]. Females also emit trills during the day of oestrus (2–4 h of fertilization phase; [50]), but more often during the formation of female sleeping groups at dawn or during mother–infant reunions (e.g. [44,47]). The adult trill call is not expressed at birth but gradually develops during infancy [51]. To date, individual-, colony- and population-specific vocal signatures in trills have been reported only for males [46,52–54]. Thereby, Hafen *et al*. [46] reported an effect of familiarity on male trill calls while controlling for genetic markers and morphometric similarity. However, Kessler *et al*. [54] showed a correlation between acoustic dissimilarity and patrilineal but not matrilineal genetic dissimilarity. For female grey mouse lemurs, individual and group vocal signatures have not been investigated yet.

The aims of this study are to investigate whether social accommodation exists in grey mouse lemurs and whether sex differences in social accommodation owing to the sex-specific social lifestyle can be found. Therefore, we investigate (i) sex differences between the trills, (ii) the existence of individual signatures in the trills of both sexes, and (iii) whether the acoustic dissimilarity between two individuals of the same sex correlates with genetic relatedness or social contact time. If social accommodation takes place in grey mouse lemurs, we expect that acoustic dissimilarity should correlate with social contact time, independent of genetic relatedness and the body weight dissimilarity score used as a proxy for anatomical differences.

## 2. Material and methods

### (a) Dataset

The dataset consisted of 482 adult grey mouse lemur trills from 36 adult grey mouse lemurs (22 females, 14 males; electronic supplementary material, table S1) and originated from the sound archive of the Institute of Zoology, University of Veterinary Medicine Hannover, Germany. The animals were housed in the captive self-sustaining grey mouse lemur colony of the institute (housing licence no.: 42502/1TiHo) in groups of two to three adult subjects or families of a mother and one to four infants per cage. Group composition was based on management decisions (for housing details, see Wrogemann *et al*. [55]). The trills were recorded during social encounter and mother–infant reunion experiments conducted between 2003 and 2012. Details on the recording procedure and equipment can be found in the electronic supplementary material, table S2. The studies were performed in accordance with the German and European regulations concerning the protection of experimental animals valid for the respective year (experimental licence no.: 33.9-42502-05-11A117; 33. 12-42502-04-14/1454).

### (b) Acoustic measurements

**Figure 1.**
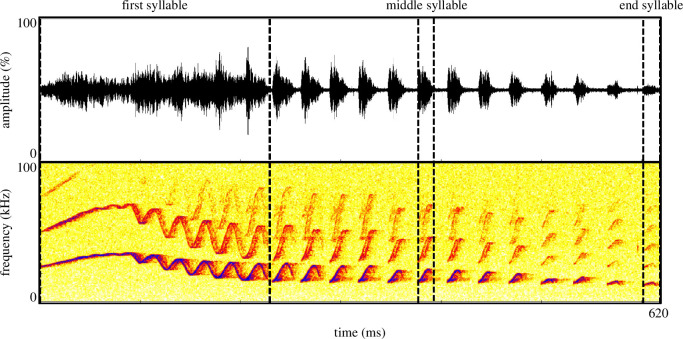
Example of a grey mouse lemur trill call. The first, middle and end syllables, whose acoustic parameters were used in the analyses, are annotated.

A trill call (figure 1) consists of up to 30 syllables, which are distinguished into three structural parts: (i) the first part, a variable initial modulation; (ii) the middle part comprising down- and/or up-modulated syllables; and (iii) the end part consisting of syllables of almost constant frequency [46,56]. Therefore, the acoustic measurements were conducted on the whole trill call and on syllable level, with a syllable being defined as the smallest vocal unit of continuous sound energy. To be consistent across individuals, the description of the tempo-spectral structure of the trill syllables was restricted to the acoustic parameters of the first, middle and end syllables of the trills, representing the three main structural parts of the trill. The first and end syllables corresponded to the beginning and end of a trill. The number of the middle syllable was denoted as half of the total number of syllables in a trill. In the case of non-whole numbers (e.g. 9.5), the result was rounded up (e.g. 10). In total, 29 acoustic parameters were measured for each trill (duration and number of syllables for the whole trill call and nine parameters each for the first, middle and end syllables of a trill call (*N*_syllables_ = 1446; definitions of the acoustic parameters can be found at the electronic supplementary material, table S3). The acoustic parameters were measured using a custom-written Script in Praat (v. 5.4.04; Phonetic Sciences, University of Amsterdam, Netherlands [57]).

### (c) Testing for sex differences and individual signatures

To test for sex-specific differences in the acoustic parameters, linear-mixed effect models (LMEs) were calculated using the acoustic parameters as the test variables and sex as the predictor variable while controlling for individuals. To investigate whether trills can be classified by sex, a nested permuted discriminant function analysis (pDFA; [58]) was performed using sex as a test factor while controlling for individual. To meet the assumption of independence of the acoustic parameters, principal component analysis (PCA) was carried out beforehand and the pDFA was calculated using the extracted factors (principal components (PCs)) with an eigenvalue greater than 1. The PCA was performed in SPSS (v. 29.0.0.0 (241)), while the other calculations were carried out in R (v. 4.0.5.; 2021-03-31 [59]; accessed using the integrated development environment RStudio (v. 1.4.1106 [60]).

To test for individual differences in the acoustic parameters of the trill call, we calculated a MANOVA using the acoustic parameters as a dependent variable and the individual as a fixed factor for each sex. To confirm the existence of individual vocal signatures in female and male trills of grey mouse lemurs, independent discriminant function analyses (DFAs) using the leave-one-out method for cross-validation combined with PCAs were applied. Thus, we first performed PCAs for both sexes separately. Then, with the extracted PCs with an eigenvalue higher than 1, the DFAs were calculated. To test whether the results were above the chance level, we performed a binomial test for each individual on the original classification results. To compare the level of individual distinctiveness across sexes, we calculated the level of agreement using Cohen’s kappa coefficient [61] and the information criterion [62,63]. Both methods are reliable when comparing datasets of different sample sizes [61–63]. The calculations were carried out using SPSS (v. 29.0.0.0 (241)), R (v. 4.0.5.; 2021-03-31 [59]; accessed using the integrated development environment RStudio (v. 1.4.1106 [60]) and Excel (v. 2016).

### (d) Acoustic dissimilarity, genetic relatedness, social contact and body weight dissimilarity scores among individuals

The acoustic dissimilarity score was calculated using Euclidean distances as a measurement of acoustic dissimilarity between individuals. Beforehand, the median for each acoustic parameter per individual was calculated, and these data were standardized using the *z*-score. The matrix was standardized to a range from 0 to 1 using the min–max normalization, with the values ranging from 0 (identical) to 1 (mostly different). The *z*-score and the Euclidean distance were calculated in R (package: stats—‘dist’ (v. 4.0.5)).

The genetic relatedness score was calculated as an autosomal kinship matrix using the breeding colony records maintained since the founding of the colony in 1985 [54]. The kinship coefficient between two individuals describes the likelihood that a randomly chosen allele at a given locus would be identical by descent, i.e. inherited from a common ancestor. The values ranged from 0 (unrelated) to 0.5 (identical). In some cases, subjects could achieve values above 0.5 owing to inbreeding in the colony. The autosomal kinship matrix was calculated in R (package: kinship2—‘kinship’ (v. 1.9.6) [64]).

The social contact score between individuals was calculated as the time the individuals had spent with each other in the same cage before the respective recordings were taken. For each individual, it was noted how many days they spent sharing a cage (a social partner) with the other individuals using the colony records concerning the housing of the animals. The matrix was standardized to a range from 0 (never together) to 1 (always together) using the min–max normalization. Since the available recordings were not recorded on the same days for all individuals, the sociality matrix was not symmetrical.

The body weight dissimilarity score was calculated based on the weekly body weight recordings of the whole colony. Body weight was used as a proxy for morphometry. The body weight dissimilarity score was calculated similarly to the acoustic dissimilarity score.

### (e) Comparisons between matrices

To investigate the effect of genetic relatedness, social contact and body weight on the individual vocal signatures, LMEs were performed for each sex using the acoustic dissimilarity score as a test variable and genetic relatedness, social contact and body weight dissimilarity scores, as well as an interaction between the three main terms, as predictor variables while controlling for the individuals of the dyads as a random factor. Since the recordings of the subjects were done in different years, there could be a time difference between the recordings of dyad partners of up to 9 years. To control for the fact that this time difference did not affect our results on sociality owing to potential changing cage partners, we added time difference (recording year of individual 1 − recording year of individual 2) and the interaction between social contact score and time difference as an additional predictor term (full model: acoustic dissimilarity score ~ genetic_relatedness * social_contact * body_weight + social_contact * time_difference, random =~1|individual1/individual2). The LMEs were conducted using the backward elimination procedure to determine the minimum adequate model (final model) [65]. The model selection was carried out by removing at each step the highest-level interaction with the highest non-significant *p*‐value. In addition, after each elimination, the previous model was compared to the reduced model by means of Wald test statistics. The elimination procedure was stopped when (i) only the main terms remained in the final model, (ii) the remaining interaction terms had a significant *p*‐value, or (iii) the Wald test statistics revealed a significant difference between the reduced and the previous model. The models were fitted by maximizing the log-likelihood. We report only final models. The calculations were conducted in R (package: nlme—‘lme’ (v. 3.1-152)).

To ensure that the bias of females being primarily housed with related females while males were mainly housed with unrelated males did not affect our results, we tested the effect of the social relationship of the dyads on the matrices. Therefore, we categorized socialized dyads into parent/sibling (mother/sister, father/son) or female/male dyads (not first-degree-related females and males). We then conducted LMEs using the acoustic dissimilarity, genetic relatedness or social contact score as test factors and dyad relationship as a predictor variable while controlling for the individuals of the dyads.

In addition, the acoustic dissimilarity score was compared with the genetic relatedness, social contact and body weight dissimilarity score matrices for both sexes separately using permutated Mantel tests and partial permutated Mantel tests in PASSaGE 2 (v. 2.0.11.6 [66]; number of permutations = 999). Since the social contact score had a high number of zeros (individuals were never housed together), which might dampen the effect of sociality, we further performed Pearson correlations between acoustic dissimilarity and social contact score only for dyads that had contact with each other (social contact score >0).

## 3. Results

### (a) Sex-specific and individual vocal signatures

Sex-specific differences in the acoustic parameters of the trills were found for 15 out of 29 acoustic parameters (*t* ≥ |2.083|, d.f. = 1, *p* ≤ 0.045; electronic supplementary material, table S4 and figure S1). The PCA extracted eight PCs with an eigenvalue higher than 1, explaining 87.72% of the variance (electronic supplementary material, table S5). Conducting a nested pDFA using the extracted eight PCs, 83.50% of the trills were correctly classified to the respective sex (versus 67.11% chance level, *p* = 0.001; cross-validation: 79.84% versus 60.18% chance level, *p* < 0.001).

Comparing the level of individual distinctiveness between sexes, we performed MANOVAs and independent DFAs in combination with PCAs for each sex separately. For both sexes, almost all acoustic parameters differed significantly between individuals (females: *F* ≥ 5.07, d.f. = 21, *p* < 0.001 except timeminF0_E; males: *F* ≥ 4.43, d.f. = 13, *p* < 0.001 except duration_M and timeminF0_M). The PCAs for both sexes extractedeight PCs with an eigenvalue greater than 1, explaining 86.43% of the variance for the females and 90.14% of the variance for the males (electronic supplementary material, table S6). The independent DFAs correctly assigned 66.5% of the female trills (cross-validation: 59.5%, chance level ≤ 7.0%) and 89.4% of the male trills (cross-validation: 82.8%, chance level <10.1%) to the respective sender. Thereby, all male individuals (binomial tests: *p* < 0.001) and 19 out of the 22 females (binomial tests: *p* < 0.001) were correctly classified above the chance level in the original classification. Males showed higher values for Cohen’s kappa coefficient and the information criterion in comparison to females (males: *κ* = 0.885 indicating almost perfect agreement; *H*_*s*_ = 8.579; females: *κ* = 0.647 indicating substantial agreement; *H*_*s*_ = 6.467), suggesting a higher level of individual distinctiveness in males compared to females. Examples of trills from different individuals for both sexes can be found in electronic supplementary material, figure S2.

### (b) Acoustic dissimilarity versus genetic relatedness, social contact and body weight dissimilarity scores

A sex-specific impact of social contact and genetic relatedness on the acoustic dissimilarity score was revealed by the LMEs. For both sexes, the final models contained only the main terms. For females, the acoustic dissimilarity score was significantly affected by social contact and body weight dissimilarity scores (*t* ≥ |2.731|, d.f. = 436, *p* ≤ 0.007; table 1) but not by genetic relatedness score or time difference (*t* ≤ |0.992|, d.f. = 436, *p* ≥ 0.322; table 1). In contrast, for males, acoustic dissimilarity was affected by genetic relatedness and body weight dissimilarity scores (*t* ≥ |3.310|, d.f. = 164, *p* ≤ 0.001; table 1) but not by social contact and time difference (*t* ≤ |1.752|, d.f. = 164, *p* ≥ 0.082; table 1).

**Table 1.** Results of the final models of the LMEs testing the effects of social contact, genetic relatedness, body weight dissimilarity and time difference scores of the dyads (predictor variables) on the acoustic dissimilarity score (test variable), while controlling for both individuals of the dyads (random factor). s.e. = standard error, d.f. = degrees of freedom; bold: *p* < 0.05.

factor	estimate	s.e.	*t*-value	*p*‐value
**females (d.f. = 436)**				
social contact	−0.190	0.067	−2.849	**0.005**
genetic relatedness	−0.124	0.125	−0.992	0.322
body weight	0.085	0.031	2.731	**0.007**
time difference	−0.002	0.002	−0.729	0.467
**males (d.f. = 164)**				
social contact	0.107	0.064	1.669	0.097
genetic relatedness	−0.577	0.152	−3.790	**<0.001**
body weight	0.132	0.040	3.310	**0.001**
time difference	0.010	0.006	1.752	0.082

**Figure 2.**
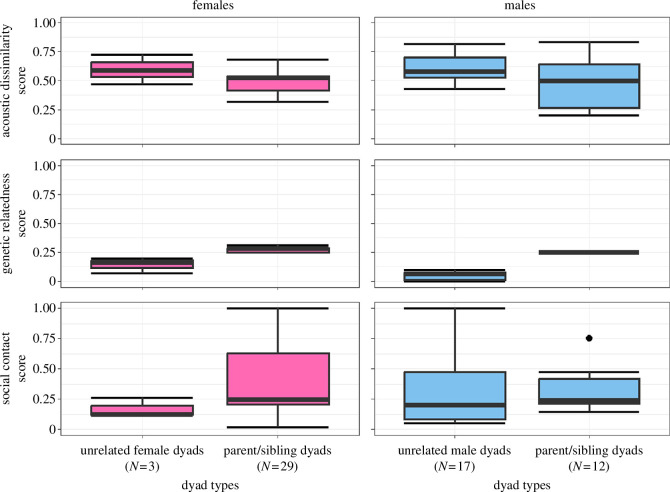
Comparisons of acoustic dissimilarity, genetic relatedness and social contact score for the individuals of both sexes that had socialized with each other. Boxplots represent the median (black line), interquartile range (box) and non-outlier range (whiskers). *N* = number of dyads.

Investigating socialized dyads confirmed the sex-specific differences, with differences in social contact score being ruled out as a driving factor (electronic supplementary material, table S7; figure 2). Thus, parent/sibling dyads did not differ from genetically less related female/male dyads in the social contact score for both sexes (females: *t* = 1.316, d.f. = 13, *p* = 0.211; males: *t* = −0.204, d.f. = 15, *p* = 0.841). Nevertheless, males showed a trend for a higher acoustic dissimilarity score for male dyads compared to father/son dyads (*t* = −1.979, d.f. = 15, *p* = 0.067), whereas mother/sister dyads did not differ from female dyads (*t* = −1.602, d.f. = 13, *p* = 0.133; electronic supplementary material, table S7).

**Figure 3.**
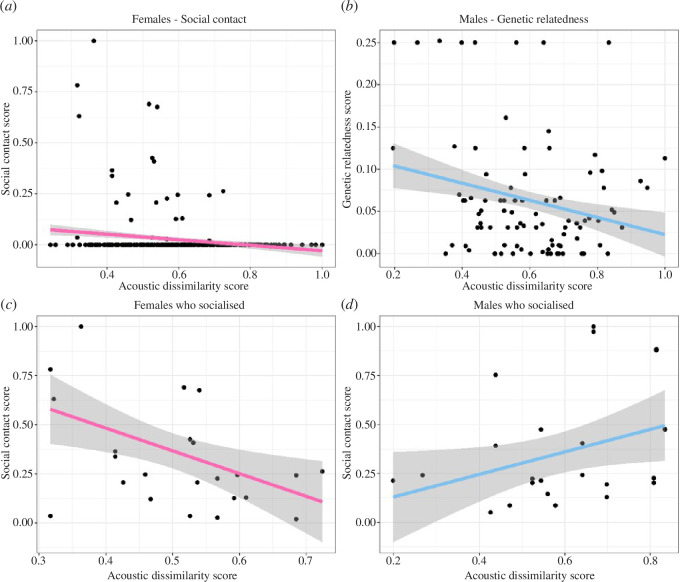
Relationship between the acoustic dissimilarities of adult trills with the social contact score and genetic relatedness for both sexes. (*a*) Relationship between acoustic dissimilarity and social contact score for females. (*b)* Relationship between acoustic dissimilarity and genetic relatedness score for males. (*c,d)* Relationship between acoustic dissimilarity and social contact score for females and males who socialized with each other, respectively. Black spots—dyads, pink line—regression line for the females, blue line—regression line for the males, grey region around the lines—confidence interval (0.95).

The sex-specific impact of social contact and genetic relatedness on the acoustic dissimilarity score was also supported by Mantel tests when correlating the acoustic dissimilarity score with genetic relatedness, social contact and body weight dissimilarity scores (table 2). For the females, the acoustic dissimilarity score showed a significant negative weak correlation with the social contact score (*r* = −0.173, *p*_2-tailed_ = 0.006; figure 3*a*), while no significant correlations with genetic relatedness and body weight dissimilarity score were found (genetic relatedness score: *r* = −0.107, *p*_2-tailed_ = 0.195; body weight dissimilarity score: *r* = 0.166, *p*_2-tailed_ = 0.165). The correlation between acoustic dissimilarity and social contact score was further confirmed when the genetic relatedness (*r* =−0.139, *p*_2-tailed_ = 0.042) or body weight dissimilarity score (*r* = −0.183, *p*_2-tailed_ = 0.001) were held constant. In contrast, for males, acoustic dissimilarity correlated significantly with the genetic relatedness (*r* =−0.239, *p*_2-tailed_ = 0.006; figure 3*b*), but not with social contact or body weight dissimilarity scores (social contact score: *r* = 0.063, *p*_2-tailed_ = 0.480; body weight dissimilarity score: *r* = 0.271, *p*_2-tailed_ = 0.118). The correlation between acoustic dissimilarity and genetic relatedness score was further supported when social contact (*r* = −0.271, *p*_2-tailed_ = 0.006) or body weight dissimilarity scores (*r* = −0.230, *p*_2-tailed_ = 0.011) were held constant.

**Table 2.** Overview of the permutated Mantel and permutated partial Mantel tests comparing acoustic dissimilarity with genetic relatedness, social contact and body weight dissimilarity scores conducted for females and males separately. Bold: *p* < 0.05.

matrices tested against acoustic dissimilarity	females	males
**permutated Mantel tests**		
genetic relatedness	*r* = −0.107, *p*_2-tailed_ = 0.195	***r* = −0.239, *p***_**2-tailed**_ **= 0.006**
social contact	***r* = −0.173, *p***_**2-tailed**_ **= 0.006**	*r* = 0.063, *p*_2-tailed_ = 0.480
body weight	*r* = 0.166, *p*_2-tailed_ = 0.165	*r* = 0.271, *p*_2-tailed_ = 0.118
**permutated partial Mantel tests**		
genetic relatedness constant: social contact	*r* = −0.027, *p*_2-tailed_ = 0.773	***r* = −0.271, *p***_**2-tailed**_ **= 0.006**
genetic relatedness constant: body weight	*r* = −0.125, *p*_2-tailed_ = 0.142	***r* = −0.230, *p***_**2-tailed**_ **= 0.011**
social contact constant: genetic relatedness	***r* = −0.139, *p***_**2-tailed**_ **= 0.042**	*r* = 0.146, *p*_2-tailed_ = 0.085
social contact constant: body weight	***r* = −0.183, *p***_**2-tailed**_ **= 0.001**	*r* = 0.047, *p*_2-tailed_ = 0.572

Focusing only on dyads who had socialized with each other (social contact score >0), the negative correlation coefficient increased for females, showing a moderately significant negative correlation between social contact and acoustic dissimilarity (*r* = −0.461, *p*_2-tailed_ = 0.008; figure 3*c*). In contrast, in males, a significant positive correlation was found (*r* = 0.376, *p*_2-tailed_ = 0.045; figure 3*d*). Thus, the more time females were housed together, the more similar their trills became, while the more time males were housed together, the more dissimilar their trills became.

## 4. Discussion

Our study shows that while individual vocal signatures in the trills of both sexes exist in the grey mouse lemur, differences in social accommodation are present. For females, acoustic dissimilarity correlated negatively with the social contact score independently of genetic relatedness and body weight differences, suggesting social accommodation in the direction of vocal convergence. For males, the results depended on whether the dataset consisted of all of the males’ trills or only those from the males who had socialized with each other. When using all of the males’ trills, a significant effect of genetic relatedness on the acoustic dissimilarity was documented, with related males producing more similar trill calls, thus suggesting a lack of social accommodation. However, when the analysis was confined to males who had socialized with each other, socialized males emitted trills that were more dissimilar to each other, suggesting social accommodation in the direction of vocal divergence. Since social contact had a significant effect on the structure of the adult trill, this highlights that grey mouse lemurs are capable of social accommodation, which is affected by their sex-specific social lifestyle.

Sex differences in the trill calls of grey mouse lemurs were shown. As grey mouse lemurs lack anatomical differences between sexes [67] and no sex differences were found for two agonistic call types [68], these sex differences are probably a consequence of different call functions and context usage [47,48,69] rather than differences in their vocal apparatus. Admittedly, the recordings from the females originated mainly from mother–infant reunions, whereas the recordings from the males originated mainly from male–female encounters. However, these sex-specific contexts are in accordance with the natural sex-specific usage of trills, reflecting different functions for females and males [46,47]. For both sexes, individual distinctiveness could be shown, highlighting the existence of individual vocal signatures in the trill calls of females, while confirming previous findings for males [48,52]. Interestingly, the level of individual distinctiveness differed between the sexes, with males having a higher Cohen’s kappa coefficient and information criterion than females. This is in line with our findings that genetic relatedness had a significant effect on the acoustic structure of male trill calls, with less variation in male trills being expected. In contrast, in females, social contact score affected the trill structure, which may have resulted in a higher acoustic variability of their trill calls owing to individual social experiences.

Social accommodation could be shown for females, which converge the acoustic structure of their trill call the more time they spend with each other, independent of their genetic relatedness or body weight dissimilarity. Since our captive mouse lemurs cannot freely choose social partners, we can rule out the possibility that the similarities in the structure of their trill calls are a result of vocal differences influencing how individuals spend time with each other, thus supporting that social relationships drive social accommodation. Although females forage solitarily during night, at dawn they meet again to form kin-related female sleeping groups [39], which increase the survival of female offspring [69]. Thereby, they exchange trill calls, which are suggested to coordinate sleeping group formation [47]. Although female sleeping groups are kin-related, high genetic relatedness does not always predict sleeping groups, with related females occasionally sleeping in different sleeping groups or sleeping groups of unrelated females having been observed [38]. Therefore, the convergence of the trill calls can be highly beneficial for the females, allowing them to establish and maintain social bonds leading to sleeping group vocal signatures, which can serve as a password for group membership [16,70].

For males, the results were dependent on whether or not the dataset included only the males who had socialized with each other. Analysing all male dyads (dataset included both socialized and non-socialized dyads), the acoustic dissimilarity of the trill calls correlated with genetic relatedness but not with social contact or body weight dissimilarity scores, suggesting that male trills were not affected by sociality. However, focusing only on socialized males, we found a positive correlation between acoustic dissimilarity and social contact score, indicating that males show social accommodation by vocally diverging the more time they spend in the same cage. Admittedly, our dataset included socialized females that were often related, whereas socialized males were often unrelated owing to our housing conditions reflecting the natural social organization. However, comparing the acoustic dissimilarity between mother–daughter/father–son dyads and female/male dyads showed similar sex-specific effects even if the social contact score did not differ. This suggests that although male trills are predominantly determined by genetic factors, males are still able to diverge their calls to some degree from those of their cage partners. The combination of genetic and social information in individual vocal signatures was also found in mandrills [14], where acoustic similarity decreased with familiarity when the genetic relationship was low. This suggests that primate vocalizations can be shaped by both genetic factors and social experience. The combination of several cues might also explain why body weight affected the acoustic dissimilarity when tested using LMEs but did not interact with genetic relatedness and social contact score.

Although male mouse lemurs disperse, male dispersal is not obligatory for all males. Radespiel *et al*. [42] reported that 43% of the males stayed close to their mother’s home range (<230 m) and that already dispersed males returned in later mating seasons [42]. Therefore, encoding kinship is still relevant for mouse lemurs to avoid inbreeding. This is in accordance with the previous findings of Zimmermann & Hafen [53] and Kessler *et al*. [54], who also found correlations between acoustic structure and genetic relationship for male mouse lemurs. The fact that social experience can also affect male trill calls is in accordance with the study of Hafen *et al*. [46] in wild mouse lemurs. There, two subpopulations living in the Kirindy Forest differed acoustically but not genetically or morphologically. Interestingly, within each subpopulation of these wild grey mouse lemurs, individuals' trills were more similar to one another, whereas in our study, the trills of socialized males became increasingly different. Although this seems to contradict our results, it might be possible that the direction of social accommodation can represent different social dynamics [28], as our study was conducted at a dyadic and not at a population level. This is in line with studies in parrots, which either converge or diverge their calls to playbacks of conspecifics depending on the familiarity of the playback donor [24,25]. Furthermore, primates can also converge or diverge their vocalizations depending on the audience, e.g. neighbouring groups (e.g. Diana monkeys [71]; chimpanzees: [27,72]). Walløe *et al*. [25] suggest that when it is more important to signal individual identity than group affiliation, individuals tend to not change or to vocally diverge from conspecifics. This suggests that while grey mouse lemur trills can encode both kinship and social experience, kinship may be encoded in a more stable manner, whereas individual distinctiveness owing to social experience is affected by a changing social environment and the function of the call. Thus, when facilitating group formation, trills converge, whereas trills used for mate attraction diverge.

To conclude, social accommodation ability was demonstrated in grey mouse lemurs, with different results for females and males. Whereas female grey mouse lemurs showed social accommodation in the direction of vocal convergence, the acoustic structure of male trill calls was mainly affected by genetic factors but also showed an underlying effect of social experience, with socialized males diverging the acoustic structure of their advertisement calls from their cage partners. The sex-specific findings may be a consequence of the different social lifestyles of the sexes reflected in different functions of the trills. Females use trills for social cohesion, whereas males use them as advertisement calls, where the effect of genetics is more prominent than that of social experience. The function as advertisement call may also explain the stronger impact of genetic relatedness to signal kinship but also the vocal divergence between cage partners to pronounce individual differences to potential mates. Playback studies further support the recognition of genetic relatedness in recipients of male trills to coordinate inbreeding avoidance [54], with further experimental approaches using translocation being important to investigate the impact of the social function of the trill calls on sex-specific social accommodation under controlled conditions. Nonetheless, our results suggest that social accommodation is not only restricted to pair-bonded and group-living mammals but can also occur in dispersed-living mammalian species when sufficient auditory input is present for the adaptation of vocalizations to those of conspecifics [16]. Thus, depending on the function of the call type, social accommodation can be important in coordinating social interactions within and between groups (group cohesion versus mating).

## Data Availability

The dataset supporting this article is available in Zenodo repository at [73]. Video and audio files are stored in the sound archive of the Institute of Zoology, University of Veterinary Medicine Hannover and are available on reasonable request from Dr Marina Scheumann. Electronic supplementary material is available online [74].
